# Starvation Protects Hepatocytes from Inflammatory Damage through Paradoxical mTORC1 Signaling

**DOI:** 10.3390/cells12121668

**Published:** 2023-06-20

**Authors:** Iqra Hussain, Harini K. Sureshkumar, Michael Bauer, Ignacio Rubio

**Affiliations:** 1 Department for Anesthesiology & Intensive Care Medicine, Jena University Hospital, Member of the Leibniz Center for Photonics in Infection Research (LPI), 07747 Jena, Germany; iqra.hussain@med.uni-jena.de (I.H.); harini.sureshkumar@uni-jena.de (H.K.S.); michael.bauer@med.uni-jena.de (M.B.); 2 Integrated Research and Treatment Center, Center for Sepsis Control and Care, Jena University Hospital, 07747 Jena, Germany

**Keywords:** AMPK, sepsis, mTORC2, calorie restriction

## Abstract

Background and aims*:* Sepsis-related liver failure is associated with a particularly unfavorable clinical outcome. Calorie restriction is a well-established factor that can increase tissue resilience, protect against liver failure and improve outcome in preclinical models of bacterial sepsis. However, the underlying molecular basis is difficult to investigate in animal studies and remains largely unknown. Methods: We have used an immortalized hepatocyte line as a model of the liver parenchyma to uncover the role of caloric restriction in the resilience of hepatocytes to inflammatory cell damage. In addition, we applied genetic and pharmacological approaches to investigate the contribution of the three major intracellular nutrient/energy sensor systems, AMPK, mTORC1 and mTORC2, in this context. Results: We demonstrate that starvation reliably protects hepatocytes from cellular damage caused by pro-inflammatory cytokines. While the major nutrient- and energy-related signaling pathways AMPK, mTORC2/Akt and mTORC1 responded to caloric restriction as expected, mTORC1 was paradoxically activated by inflammatory stress in starved, energy-deprived hepatocytes. Pharmacological inhibition of mTORC1 or genetic silencing of the mTORC1 scaffold Raptor, but not its mTORC2 counterpart Rictor, abrogated the protective effect of starvation and exacerbated inflammation-induced cell death. Remarkably, mTORC1 activation in starved hepatocytes was uncoupled from the regulation of autophagy, but crucial for sustained protein synthesis in starved resistant cells. Conclusions: AMPK engagement and paradoxical mTORC1 activation and signaling mediate protection against pro-inflammatory stress exerted by caloric restriction in hepatocytes.

## 1. Introduction

Sepsis refers to a severe infection syndrome that manifests in heterogeneous clinical pictures and is characterized by the failure of one or more organs in the course of infection [1]. In some scenarios, the pathogen itself causes direct organ damage, e.g., by releasing cytotoxins, while in other cases, organ failure may occur as “collateral damage” of an inadequate host response [2], e.g., in the context of an exaggerated hyper-inflammatory burst commonly referred to as a “cytokine storm” [3]. 

While liver failure is often considered to occur only late in sepsis, its consequences are particularly devastating and associated with a sharp increase in sepsis-related mortality [4,5]. This prominent role is probably related to the liver’s pivotal systemic role in metabolic supply, detoxification and host defense [6]. For example, disruption of hepatic gluconeogenesis leads to a decline in glucose supply during severe infections, exacerbating sepsis and sepsis-related mortality in mice [7]. Preventing organ damage in general and preventing liver failure in particular are therefore important goals in the clinical management of sepsis.

One intervention that has recently attracted much attention as a potential way to strengthen organ resilience to infection is caloric restriction and the associated changes in energy expenditure. Fasting protects against bacterial sepsis in mice [8], and excessive calorie intake reinforces liver damage in human sepsis [9]. Interestingly, the protection afforded by caloric restriction can be observed in purified primary hepatocytes [10], suggesting that dietary cues are sensed and processed directly by the hepatocytes.

Due to its central role as the metabolic hub of the organism, the liver is particularly sensitive to external signals triggered by metabolic cues, including hormones (insulin, glucagon) or the availability of nutrients. Sophisticated intracellular signaling networks recognize and process signals elicited by hormones, nutrients and the intracellular energy status. These cues are mainly funneled via the metabolic sensor kinases AMP-activated protein kinase (AMPK) and mammalian target of rapamycin (mTOR). AMPK is a heterotrimeric Ser/Thr master kinase that senses an increase in the intracellular AMP/ATP ratio by directly interacting with AMP via its γ−subunit [11,12]. Once bound to AMP, AMPK is locked into its active conformation by phosphorylation of Thr172 in the ß-subunit. Active AMPK in turn induces a plethora of downstream effects, including a boost of energy-generating metabolic pathways (e.g., glycolysis) and inhibition of energy-consuming processes such as cholesterol or fatty acid synthesis by inhibiting the corresponding rate-limiting enzymes 3-hydroxy-3-methyl-glutaryl-coenzyme A (HMG-CoA) reductase or acetyl-CoA carboxylase [13,14]. Work with AMPK knockout mice indicated a hepatoprotective role of AMPK in sepsis [15,16], suggesting that dietary signaling via AMPK may contribute to the adaptation and/or resilience of liver tissue to settings of severe infections. 

A second intracellular metabolic pathway that senses and responds to intracellular energy supply as well as extracellular hormonal signals and nutrient availability is named after its core Ser/Thr-kinase mTOR. mTOR exists as part of two related, yet functionally different multiprotein complexes: mTORC1, built around the scaffold protein Raptor, and mTORC2, assembled around the scaffold protein Rictor. The eponymous macrolide rapamycin can be used to distinguish between the two complexes, as short-term treatment with rapamycin inhibits mTORC1, but not mTORC2 [17]. Increasing evidence suggests that mTORC2 is identical to 3-phosphoinositide-dependent protein kinase-2 (PDK2), the kinase responsible for the activating phosphorylation of Akt, also known as protein Kinase B (PKB), at Ser473 [18,19]. However, much more is known about the function and regulation of mTORC1. mTORC1 funnels and integrates signals from hormones and growth factors, nutrients (amino acids) and the cellular energy status to become fully activated. Active mTORC1 in turn activates protein and lipid biosynthesis and inhibits catabolic pathways such as autophagy [17,20,21]. Given the mirror response of both pathways to resource abundance (mTORC1 high; AMPK low) and resource scarcity (AMPK high; mTORC1 low), the two pathways commonly trigger opposite reactions in cell metabolism [22]. In fact, both pathways negatively regulate each other; Thus, AMPK can block mTORC1 activity by phosphorylating the negative mTORC1-regulator tuberous sclerosis 1/2 (TSC1/TSC2) complex, thereby stimulating catabolic processes [23,24,25].

Given the reported protective role of caloric restriction in liver and hepatocytes against inflammatory insults, we were interested in delineating the contribution of the different nutrient/energy-sensing pathways mTORC1, mTORC2 and AMPK in this context. We report here an unexpected and paradoxical activation of the mTORC1 pathway in starved hepatocytes exposed to inflammatory cytokines, which resulted in protection against inflammatory cell damage. 

## 2. Materials and Methods

### 2.1. Materials

OnTarget plus siRNA smart pools were from Dharmacon/Horizon Discovery, Waterbeach, UK. Individual mice cytokines were purchased from Immunotools, Friesoythe, Germany, and LPS was purchased from Invitrogen/Thermo Fischer Scientific, Waltham, MA, USA. Propidium iodide was from Calbiochem/Merck, Darmstadt, Germany, and Hoechst33342 from Thermo Fischer Scientific, Waltham, MA, USA. Rapamycin was bought from Hycult Biotech, Uden, Netherlands, AKT inhibitor VIII from MedChem Express, Monmouth Junction, NJ, USA and cycloheximide from Merck, Darmstad, Germany. L-Azido-homo-alanine (L-AHA) and Protein synthesis detection Kit were from JenaBioscience, Jena, Germany. Real-time Glo apoptosis/necrosis kit was purchased from Promega, Fitchburg, USA. Insulin, holo-transferrin, sodium selenite and dexamethasone were from Sigma-Aldrich/Merck, Darmstadt, Germany. Lipofectamine RNAimax reagent was purchased from Invitrogen. BCA assay kit was from Serva Electrophoresis GmbH. ECL-substrate solution was from Thermo Fischer Scientific. DAPI-containing fluoroshield mounting medium was from Sigma-Aldrich/Merck, Darmstadt, Germany. Cyto ID autophagy kit was purchased from Enzo life sciences, Lörrach, Germany.

### 2.2. Antibodies 

Antibodies used for Western blot were purchased from Cell signaling technology, Danvers, MA, USA: AKT (pan) (C67E7), phospho-S473-AKT (D9E), phosphor-T308 AKT (244F9), AMPKα, phospho-T172-AMPKα (40H9), p70-S6K, phospho-T389-p70-S6K, S6P (5G10), phospho-S235/236-S6P, Raptor (24C12), Rictor (53A2), elF2α, phospho-S51-elF2α (119A11), ULK1 (D8H5), phospho-S757-ULK1, phospho-S555-ULK1 (D1H4), 4-EBP1 and LC3B (D11) XP. Antibodies used for immunocytochemistry: elF3η (C-5) was purchased from Santa Cruz Biotechnology, Dallas, TX, USA, and G3BP1 was purchased from Thermo Fischer Scientific, Waltham, MA, USA.

### 2.3. Cell Culture, Cell Stimulations and Treatments

AML12 immortalized mouse hepatic cells were provided by Dr. Jürgen Sonnemann (Jena, Germany). AML12 cells were maintained in DMEM supplemented with 10% fetal calf serum (FCS), 10 µg/mL insulin, 5.5 µg/mL transferrin, 5 ng/mL sodium selenite and 40 ng/mL dexamethasone under standard conditions. For starvation, cells were cultured in DMEM without glucose, sodium pyruvate, FBS and with supplements. For inflammatory stress, cells were exposed to a cytokine/LPS mix consisting of 50 ng/mL TNF, 10 ng/mL IL1β, 10 ng/mL IFNγ, 10 ng/mL IL6 and 100 ng/mL LPS. Cells were pre-treated with 500 μM AICAR (Hycultec, Beutelsbach, Germany) or 10 μM MHY1485 (Sigma-Aldrich/Merck, Darmstadt, Germany) 2 h prior to treatment with cytokines.

### 2.4. Cell Death Assay and Quantification

AML12 cells were seeded on collagen-coated self-made glass-bottom cell culture dishes [26]. Cells, either untreated or treated, were fed with membrane-permeable live-cell nucleus stain Hoechst33342 (0.1 µg/mL) for 5 min followed by the addition of 1 µM propidium iodide (PI) and incubated for a further 5 min under standard cell culture conditions. Cell monolayers were washed with cell culture medium twice, and live-cell images were captured with a laser scanning microscope using a 455 nm laser line for H33342 and 617 nm emission wavelength for PI. Images were analyzed in Zen blue 2.5 lite software and quantified using ImageJ 1.54d. Three optical fields were analyzed per experiment for each condition. Cells positive for PI or Hoechst33342 per field were counted in ImageJ, and data were plotted using GraphPad Prism 9.2.0.

### 2.5. Real-Time Glo Necrosis/Apoptosis Assay

Cells were seeded in collagen-coated luminescence white 96-well plates. The next day, 2× detection reagent was prepared according to the manufacturer’s protocol. Then, 100 µL cell culture media supplemented with the appropriate stimulants/stressors was added and 100 µL of 2× detection reagent was pipetted in all wells, including negative and positive controls. Relative fluorescence and luminescence were recorded at the desired time interval on an EnSpire multimode plate reader.

### 2.6. siRNA Transfections

Cells were seeded in complete medium on 6-well plates one day prior to transfection. Cells were transfected with Lipofectamine RNAimax according to the manufacturer’s instructions except that 10 μL of Lipofectamine RNAimax and 1 μg siRNA were used per well. Plates were incubated under standard conditions for 48–72 h, subjected to treatments and processed as appropriate.

### 2.7. Western Blot and Immune Detection

Cell monolayers were washed once with ice-cold PBS and lysed in cold RIPA lysis buffer (50 mM TRIS-HCl pH 8.0, 150 mM NaCl, 1% Nonidet P-40, 0.5% Deoxycholate, 0.1% SDS) supplemented with protease and phosphatase inhibitors. Cells were scraped off, and extracts were cleared by centrifugation at 12,000× *g* for 20 min at 4 °C. Protein concentration was determined with the BCA method, and equal amounts of protein were subjected to SDS-PAGE separation and Western blotting using standard techniques. Membranes were commonly probed with primary antibody overnight at 4 °C under agitation in TBS-Tween supplemented with 1% BSA. After washing with TBS-Tween, incubation with appropriate secondary HRP-conjugated antibody and a second round of washing chemoluminescent signal detection were performed with the Fusion FX7 EDGE Vilber documentation system.

### 2.8. Protein Synthesis Determination

Total protein synthesis was measured by metabolic labeling with azido-substituted amino acids followed by a copper-catalyzed click chemistry fluorescence detection approach (JenaBioscience, Jena, Germany). Briefly, cells seeded in 6-well plates were fed a customized methionine-free medium (Life Technologies, Carlsbad, CA, USA) supplemented with 1 mM L-Azido homo alanine (L-AHA). Cells were harvested with accutase; the pellet was washed with PBS and fixed with 4% formalin solution for 15 min at RT. This was followed by two rounds of washing with 1% BSA in PBS and a permeabilization step with 0.25% Triton X-100 in PBS for 15 min at RT. Cell pellets were washed, and 100 µL of freshly prepared CLICK reaction solution was added according to the manufacturer’s instructions. Reactions were incubated for 30 min at RT in the dark. Cell pellets were washed with PBS twice, and fluorescence intensity was measured in a BD Accuri C6 plus flow cytometer. Results were analyzed with FlowJo v.10.7.1. A shift in APC intensity in the histogram was monitored and gated based on unstained controls. The protein synthesis inhibitor cycloheximide (CHX) was applied at 100 µg/mL as a negative control.

### 2.9. Immunocytochemistry

Cells were seeded on collagen-coated glass coverslips in 24-well plates. After the appropriate treatments/stimulations, cells were fixed with 4% formalin for 15 min at RT, followed by washing and permeabilization with 0.3% Triton X-100 in PBS for 10 min. Cells were blocked with 10% BSA in PBS for 45 min at RT, followed by overnight primary antibody incubation at 4 °C with gentle agitation. The next day, after two rounds of washing, cells were incubated with appropriate fluorophore-conjugated secondary antibodies (1:500 dilutions) in blocking solution for 1 h at RT. Coverslips were mounted on the glass slides by using mounting media and kept at 4 °C until visualization by microscopy. Confocal images were captured on an LSM900 (Zeiss, Jena, Germany) set in Airyscan mode, using a 63× oil immersion objective with an optical slice of 1 μm or less. Images were analyzed in Zen blue 2.5 lite software and quantified with ImageJ using intensity thresholds of 50 and 255 and a particle size range of 0.1 to 2.5 µm of for all samples. Values were normalized to DAPI intensity.

### 2.10. Statistical Analysis

Data were presented as mean ± SEM of independent experiments as indicated in each figure legend. Statistical analysis was performed using GraphPad Prism version 9.2.0 (GraphPad Software Inc., San Diego, CA, USA), and significance was accepted when *p* value was below 0.05. One-way ANOVA or two-way ANOVA was performed depending on whether one or two variables were being compared, respectively. Post hoc test (Tukey’s or Sidak’s) was performed when two variables were being compared. Unpaired two-tailed Student’s *t*-test with Welch correction was performed when two independent groups were being compared.

## 3. Results

### 3.1. Starvation Protects Immortalized Hepatocytes against Pro-Inflammatory Cytokine/PAMP-Induced Cell Damage 

**Figure 1:**
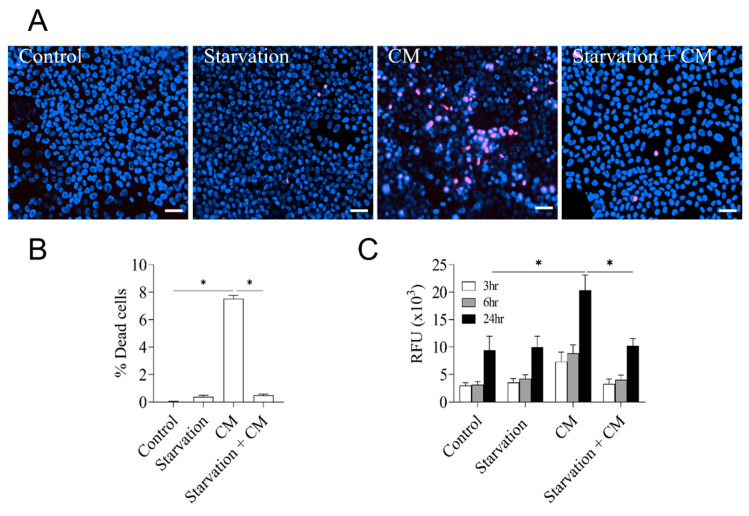
Pro-inflammatory cytokines cause cell damage and death, which is prevented by calorie restriction. (**A**) AML12 hepatocytes were exposed to the indicated stress cues and processed for the assessment of membrane integrity and cell death by dual PI/Hoescht33342 staining and live-cell imaging. Scale bar: 50 µm. (**B**) Images from (A) were analyzed and quantified in ImageJ. Results are plotted as percentage of PI-positive cells. (**C**) Cells were treated as before and subjected to a combined apoptosis and necrosis assay as described in the experimental section. The assay data are plotted as relative fluorescence units (RFUs) reflecting the loss of membrane integrity. Data are presented as mean ± SEM, one-way analysis of variance (ANOVA) followed by Tukey’s multiple comparison test. * *p* < 0.05. CM indicates a mix of pro-inflammatory cytokines and LPS. Starvation + CM: starvation followed by CM treatment.

Pro-inflammatory cytokine levels can abruptly increase systemically or locally confined in severe infections and inflict serious damage to tissue parenchyma. To mimic this scenario in an in vitro cellular model, we exposed the immortalized, non-transformed hepatocyte cell line AML12 to different combinations of cytokines and pathogen-associated molecular patterns (PAMPs). AML12 hepatocytes exhibit typical functional and structural hepatocyte features and have been widely used for metabolic and pharmacological studies. We observed that a mix of pro-inflammatory cytokines and the PAMP lipopolysaccharide (LPS), referred to from here on as cytokine mix, induced severe cell damage and extensive cell death in these cells within 24 h, as detected by direct microscopic visualization (Figure 1A, quantified in Figure 1B) or by flow-cytometric assessment of cell permeabilization (Figure 1C). These results suggest that inflammation can exert direct stress and damage hepatocytes independently of secondary systemic or immunopathological factors, in contrast to what is usually seen in hepatocellular carcinoma cells [27,28]. To investigate if a calorie/energy restriction regime (referred to also as starvation from here on) had an effect on the inflammatory damage, we removed glucose and pyruvate as the major carbon energy sources for 24 h along with the inflammatory insult. As depicted in Figure 1A–C, removing these major carbon energy sources did not compromise cell viability but exerted a strong protection from cell death elicited by the cytokine mix. These results support the idea that starvation triggers cell-autonomous programs in hepatocytes that increase their resilience to inflammatory damage.

### 3.2. Nutrient- and Energy-Sensing Pathways Respond to Starvation in Hepatocytes

**Figure 2:**
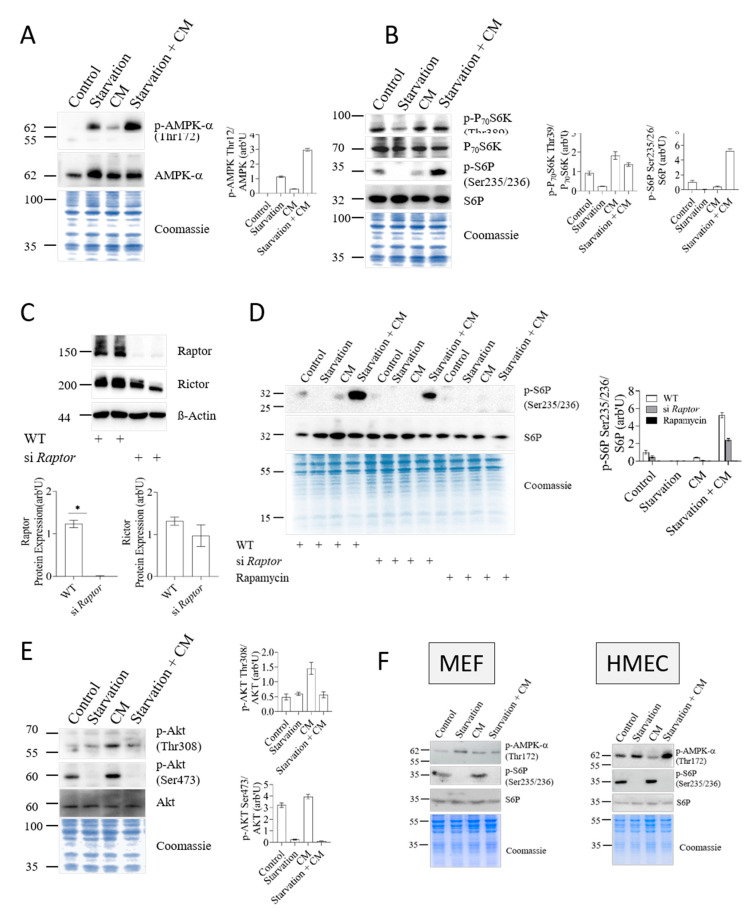
AMPKα and mTORC1 but not mTORC2 are activated in protected hepatocyte cells. Detection of AMPKα, mTORC1 and mTORC2 activity in AML12 cells. (**A**) AML12 hepatocytes were exposed to starvation, pro-inflammatory cytokines (CM) or both as described in the experimental section, and protein extracts were processed for Western blotting with the indicated antibodies. Coomassie staining of the membranes provided a total protein load control. Right panel shows the quantification of phospho-AMPKα (Thr172) performed by densitometry and two-step normalization to total AMPK levels and Coomassie lane intensity with ImageJ. (**B**) Cells were treated and processed as in panel A, followed by Western blot detection of the indicated proteins and phosphoproteins. Quantification of phospho-p70S6K (T389) and phospho-S6P (S235/S236) levels normalized to total p70S6K and S6P, respectively, is shown on the right-hand panels. (**C**) AML12 cells were subjected to siRNA-mediated knockdown of Raptor. Knockdown efficiency and selectivity were assessed by Western blotting for Raptor and Rictor. The densitometric quantification of Raptor and Rictor protein levels normalized to β-actin is plotted in the adjacent graphs. Data are presented as mean ± SEM. * *p* < 0.05. (**D**) AML12 cells were subjected to siRNA-mediated knockdown of Raptor or treated with 500 nM rapamycin for 24 h. Cell extracts were resolved by SDS-PAGE, and the levels of phosphorylated S6P (Ser235/236) or total S6P were assessed by Western blotting. Bands were quantified by densitometry, and values are plotted on the right hand as the phosphoS6P/S6P ratio additionally normalized to the Coomassie stain of total extracts. (**E**) Western blot and quantification of phospho-Akt (Thr308) and phospho-Akt (Ser473) normalized to pan-AKT and Coomassie-stained gel. (**F**) HMEC or MEF cells were exposed to starvation and/or pro-inflammatory cytokines (CM) as before and processed for Western blotting with the indicated antibodies. Membranes were stained with Coomassie to ascertain equal load. All data represent mean ± SD. CM: cytokine mix + LPS. The blots shown in (**A**,**B**) are from the same sample loading and therefore have the same Coomassie dye load control.

To understand how starvation induced protection against inflammatory damage, we measured the activity of the two main energy-sensing pathways, the AMPK and mTOR pathways. AMPK activity, as recorded by assessing phosphorylation of AMPKα on Thr172, increased in cells exposed to energy source restriction (Figure 2A), indicating that the 24 h starvation protocol had appreciably impacted the AMP/ATP ratio of hepatocytes, triggering an AMPK response. Twenty-four-hour starvation also affected the activity of the second major metabolic sensor, mTOR. As shown in Figure 2B, starvation specifically reduced the activity of mTORC1, as judged by the reduced Thr389-phosphorylation of the mTOR effector p70 ribosomal S6 kinase (p70-S6K) and Ser235/Ser236-phosphorylation of p70-S6K’s downstream substrate, the ribosomal protein S6 (S6P). Remarkably, mTORC1 activity was not reduced in response to the inflammatory insult and was even strongly increased by the inflammatory cytokine mix applied together with starvation, as judged by the Ser235/Ser236 phosphorylation state of S6P. To confirm that S6P-phosphorylation truly reflected mTORC1 activity, we knocked down the mTORC1-scaffold protein Raptor using siRNA. As depicted in Figure 2C, siRNA treatment reduced Raptor expression by 95% while Rictor levels were not significantly reduced. We speculate that the modest, if at all meaningful, decrease in Rictor protein levels was secondary to Raptor, i.e., mTORC1, downregulation. Importantly, the knockdown of Raptor or pharmacological inhibition of mTORC1 with rapamycin markedly reduced activation/phosphorylation of S6P, albeit with variable efficacy (Figure 2D). In view of the lower efficacy of Raptor siRNA versus rapamycin, we speculate that small residual amounts of Raptor protein may be sufficient to maintain S6P phosphorylation. Alternatively, rapamycin might exert effects on S6P phosphorylation via an mTORC1-independent mechanism. This notion is not unprecedented, as illustrated by the previously reported case of post-translational inhibition of nuclear factor kappa-light-chain-enhancer of activated B cells (NF-κB) [29,30]. Regardless of these considerations, we conclude from the results in Figure 2D that mTORC1 was strongly activated in starved cells exposed to an inflammatory stimulus. 

We also measured the phosphorylation/activation of Akt at Ser473 as a readout for mTORC2 activity [18]. Akt-Ser473 phosphorylation was markedly reduced following starvation (Figure 2E), irrespective of the presence of inflammatory cytokines. In contrast, the pro-inflammatory cytokine mix stimulated the phosphorylation of both major phosphorylation sites on Akt. Thus, starvation activated AMPK and inhibited both mTORC1 and mTORC2 in hepatocytes, in agreement with current concepts [31]. However, inflammatory stress triggered a seemingly paradoxical activation of mTORC1, but not mTORC2, in a background of starvation. Indeed, this effect appears to be specific for hepatocytes, as it was not observed in two unrelated non-hepatocytic cell lines (HMEC primary endothelial cells and MEF embryonic fibroblasts) (Figure 2F).

### 3.3. AMPK and mTORC1, but Not mTORC2, Mediate Starvation-Induced Protection against Inflammatory Stress 

**Figure 3:**
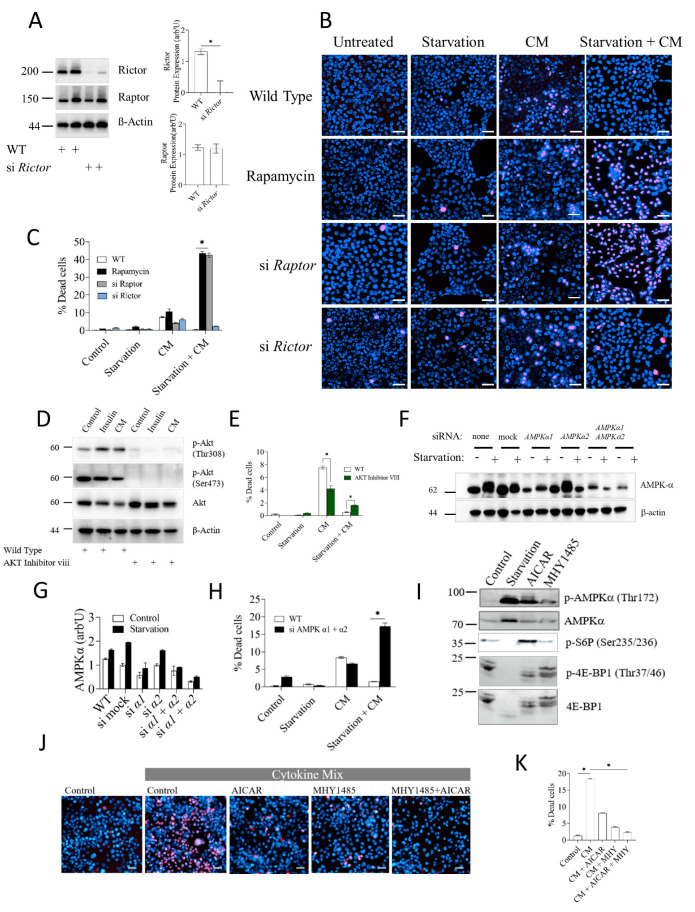
Protection against inflammatory damage provided by calorie restriction requires mTORC1 and AMPKα. (**A**) Western blot of Raptor and Rictor protein levels in cells subjected to siRNA-mediated *Rictor* knockdown. Quantification of Rictor protein levels normalized to β-actin with ImageJ is shown on the right plot. (**B**) Cell death assay in AML12 cells that underwent siRNA-mediated knockdown of *Raptor* or *Rictor*. Inflammatory stress and starvation treatments were as before. Cell death was evaluated by dual PI/Hoescht33342 live/dead staining. Scale bar: 50 µm. (**C**) Quantification of data shown in B. (**D**) Potency of Akt inhibitor VIII in AML12 cells. AML12 cells treated with 10 μM Akt inhibitor VIII for 24 h or left untreated were stimulated with insulin or cytokine mix and processed for immunodetection by Western blotting of activated/phosphorylated and total Akt with the indicated antibodies. (**E**) Cell death assay of AML12 cells treated with 10 μM Akt inhibitor VIII for 24 h or left untreated prior to exposure to starvation and/or inflammatory cytokines. (**F**) AML12 cells transfected with siRNAs against mock, AMPKα1 and/or AMPKα2 were exposed or not to overnight starvation (−/+) followed by Western blot detection of total AMPKα. None: no transfection mix or siRNA; Mock: transfection mix with control siRNA with no homology to any known gene sequence. (**G**) Densitometric quantification of AMPKα levels shown in panel (**F**). (**H**) Cell death assay of AML12 cells transfected with a mix of siRNAs against AMPKα1 and AMPKα2 and challenged by starvation and/or inflammatory cytokines as indicated. (**I**) AML12 cells were treated with AICAR or MHY1485, and cell extracts were subjected to Western blot detection with the indicated antibodies. (**J**) AML12 cells were treated with AICAR or MHY1485 and exposed to cytokine mix as indicated. Cell death was measured as before. (**K**) Quantification of cell death data in panel (**J**). Cell death data are presented as mean ± SEM; two-way analysis of variance (ANOVA) followed by Tukey’s multiple comparison test was performed. * indicates *p* < 0.05, ns indicates non-significance.

Given the strong mTORC1 activation, in the absence of mTORC2 activity, in the resilient starved hepatocyte, we questioned whether mTORC1 is necessary for starvation-induced protection against inflammatory damage. To discriminate mTORC1 and mTORC2 effects, we silenced the respective core scaffold proteins Raptor (Figure 2C) and Rictor (Figure 3A) by siRNA. As shown in Figure 3B and quantified in Figure 3C, silencing Raptor, but not Rictor, abolished the protective effect of starvation. Indeed, Raptor knockdown exacerbated the cellular damage inflicted by inflammation, while Raptor knockdown alone did not compromise cell viability. The same effects were elicited by short-term treatment with rapamycin, strongly supporting the concept that mTORC1, but not mTORC2, was necessary for starvation-induced protection.

To further support the idea that mTORC2 was not needed to confer protection, we tested the effect of inhibiting Akt, since mTORC2 is an upstream activator of Akt. Pharmacological inhibition of Akt completely abolished S473 or Thr308 phosphorylation (Figure 3D), but did not prevent the starvation-induced protection against inflammatory damage (Figure 3E). From these data, we concluded that mTORC1 but not mTORC2 is necessary for the starvation-induced protection in hepatocytes.

Since starvation triggered pronounced AMPK activation (Figure 2A), we also tested for a possible role of AMPK in starvation-dependent protection using an analogous gene silencing approach. Two isoforms of the catalytic subunit AMPKα, α1 and α2, with partially overlapping functions, are expressed in hepatocytes [12,32]. Accordingly, only the combined application of siRNAs targeting both isoforms led to a substantial decline in total AMPKα protein levels, as shown in Figure 3F and quantified in Figure 3G. Notably, silencing AMPKα1 and α2 aborted the protection exerted by starvation (Figure 3H), virtually mimicking the effect of mTORC1 inhibition. We concluded that the nutrient- and energy-sensitive kinases AMPK and mTORC1, but not mTORC2, are critical for the protective effect of starvation in hepatocytes. To understand if activation of the AMPKα or mTORC1 pathway was sufficient to protect from inflammatory stress, we employed a pharmacological approach; The AMP analog AICAR and the triazine derivative MHY1485 activate the AMPK and mTORC1 pathways, respectively. As confirmed by Western blotting from AML12 cell extracts (Figure 3I), MHY1485 induced a modest but consistent stimulation of mTORC1 signaling, best seen at the level of 4E-BP1 phosphorylation. AICAR induced strong activation of AMPK as monitored by phosphorylation on Thr172 and a concomitant mobility shift. Strikingly, AICAR also elevated the phosphorylation of the mTORC1 downstream S6P protein, evidencing again that the mTORC1 pathway is unconventionally wired in the hepatocytes. Notably, overnight treatment with any of the two drugs exerted significant protection against inflammatory cell death (Figure 3J), indicating that activation of either pathway was sufficient to exert the effect.

### 3.4. High Autophagy Rate Does Not Protect Hepatocytes from Inflammatory Damage

**Figure 4:**
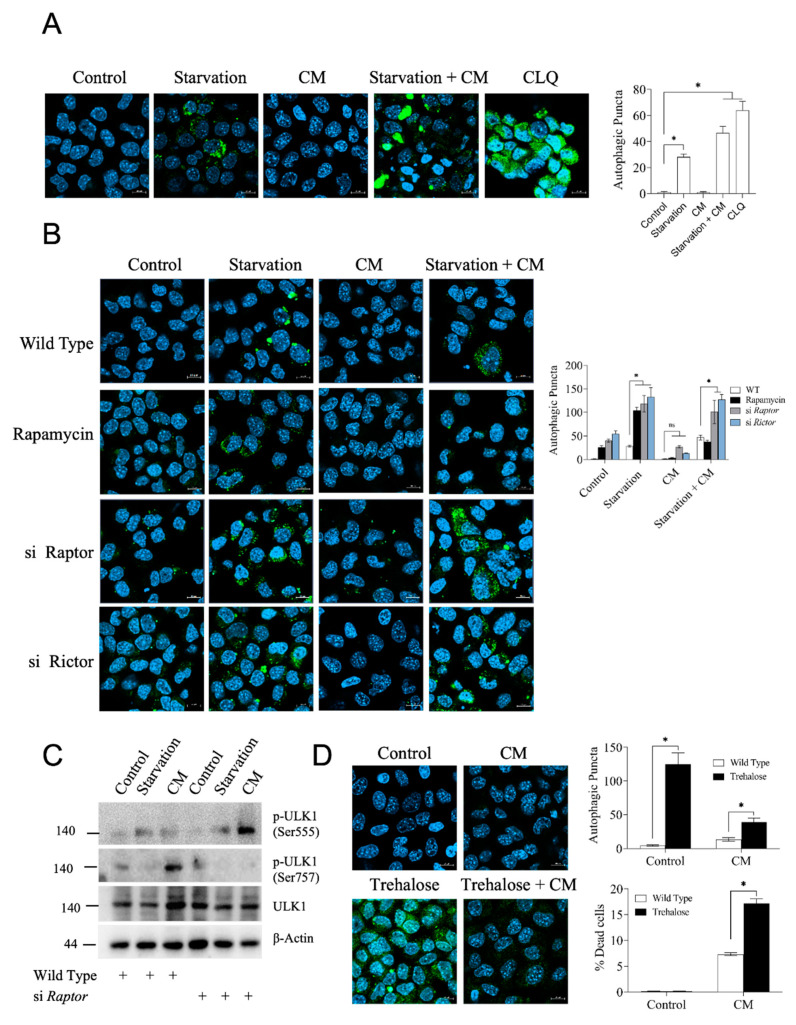
Starvation induces autophagy in hepatocytes. (**A**) Immunocytochemical assessment of autophagy in AML12 hepatocytes; see experimental section for details. Scale bar: 10 µm. Quantification of autophagic puncta with ImageJ is shown on the right-hand plot. Chloroquine (CLQ, 10 μM) was used as a positive control for autophagy induction. (**B**) Autophagy induction in AML12 cells treated with siRNA against *Raptor* or *Rictor* or treated with rapamycin prior to being challenged by a cytokine mix and/or starvation. Quantification of autophagic punctae is shown on the right panel. Scale bar: 10 µm. (**C**) AML12 cells were subjected to siRNA-mediated knockdown of Raptor where indicated and treated as before. Cell lysates were processed via Western blotting for detection of phosphorylated and total ULK-1. See text for details. (**D**) Visualization of autophagic puncta using the cyto-ID assay in cells exposed to trehalose or left untreated and challenged or not with the cytokine mix (CM). Quantification is plotted on the right. Data are presented as mean ± SEM; two-way analysis of variance (ANOVA) followed by Tukey’s multiple comparison test was performed. * *p* < 0.05; ns indicates not significant. CM indicates a mix of cytokines and LPS.

Inflammation resulted in the simultaneous activation of AMPK and mTORC1 signaling in hepatocytes subjected to a short-term energy restriction. This was remarkable because AMPK downregulates mTORC1 signaling, as both pathways usually respond in opposite ways to the same metabolic cue. Autophagy is a cellular program triggered, among other factors, in response to starvation and serves to degrade and recycle cellular constituents. Proper functioning of autophagy is important for cellular homeostasis, and high autophagy has been associated with improved tissue resilience and disease tolerance to multiple stressors, including severe inflammation and sepsis [33,34]. Hence, we wondered whether autophagy qualified as the cellular cue that mediated the starvation-induced, mTORC1-dependent resilience against inflammatory stress. Assessing autophagy by the visualization and quantification of autophagosomal puncta showed an induction of autophagy in starved cells but not in cells exposed to pro-inflammatory cytokines only (Figure 4A). This suggested that autophagy could potentially be an important component of the starvation-dependent protection. To test if autophagy mediated the mTORC1-dependent effects of protection, we measured autophagy in hepatocytes following siRNA-mediated gene silencing of Raptor or Rictor. As documented in Figure 4B, both Raptor and Rictor knockdown caused an increased occurrence of autophagic puncta, consistent with previous findings [35,36]. Since Raptor knockdown, i.e., mTORC1 ablation, prevented starvation-induced protection (see Figure 3B above), these findings strongly indicated that autophagy is not a dominant pathway involved in hepatocyte resilience acquisition. To prove these findings with an alternative approach, we assessed the activation status of Ulk1, a rate-limiting factor at the top of the autophagic regulatory cascade. Ulk1 activity and hence autophagic flux is largely dictated by the Ulk-1 phosphorylation status, with Ser555 phosphorylation by AMPK being activating, whilst Ser757 phosphorylation inhibits Ulk1-dependent nucleation of autophagy [36,37,38]. We found that starvation and exposure to pro-inflammatory cytokines exerted opposite effects on Ulk1 Ser555 vs. Ser757 phosphorylation (Figure 4C), which were fully consistent with the immunocytochemically detected rate of autophagy. In particular, knocking down Raptor, and thus mTORC1, fully inverted the autophagic response to inflammatory stress, leading to a strong increase in phosphorylation of Ser555 and inhibition of phosphorylation of Ser757 in the pro-inflammatory background, consistent with the observed strong increase in autophagy (Figure 4B). Together, these findings suggested that autophagy is not the pathway mediating starvation-induced, mTORC1-mediated protection in hepatocytes. However, since autophagy manipulation required inducing changes in mTORC1 activity, which can have multiple other consequences in cell homeostasis, we tested the effect of trehalose, a substance that strongly induces autophagy without affecting mTORC1 activity [39]. Consistent with the above notion, exposure to trehalose caused a strong induction of autophagy in hepatocytes, yet it did not protect against pro-inflammatory cell death (Figure 4D). Overall, our results suggest that autophagy, at least by itself, does not play a dominant role in the induction of hepatocyte resilience to an acute pro-inflammatory insult.

### 3.5. Protein Synthesis Rate Correlates with Cell Viability and Is Strongly Dependent on mTORC1

**Figure 5:**
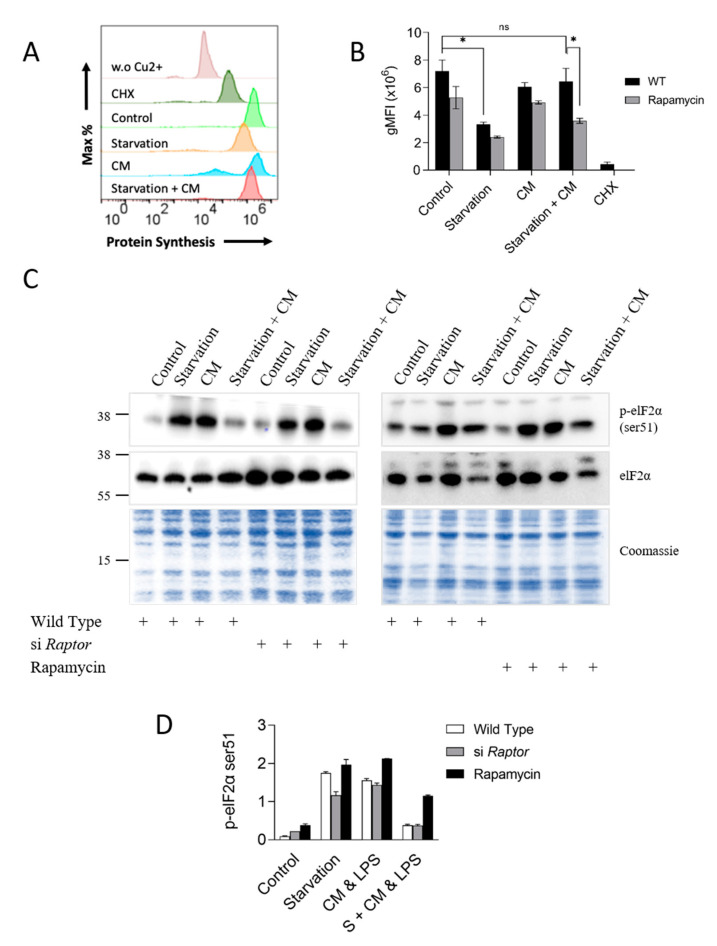
Protein synthesis rate correlates with the state of resilience in starved hepatocytes exposed to pro-inflammatory cytokines. (**A**) Histogram from flow cytometry analysis of protein synthesis in AML12 cells exposed to various treatments. (**B**) Quantification of flow cytometric data in terms of mean fluorescence intensity per 20,000 events showing protein synthesis. Data were analyzed and quantified in FlowJo and the BD Accuri software. This blot originates from the same sample loading as Figure 2E and therefore shares the same Coomassie dye load control. (**C**) Western blot analysis for phospho-elF2α (ser51) and total eIF2α in cells treated as before. (**D**) Densitometric quantification of protein bands from panel C, plotted as phospho-elF2α (ser51)/elF2α ratio, additionally normalized to Coomassie intensity of the corresponding lanes. Data are presented as mean ± SD; two-way analysis of variance (ANOVA) followed by Tukey’s multiple comparison test was performed. * indicates *p* < 0.05, ns indicates non-significance. CHX: cycloheximide. w.o. Cu^2+^:Cu^2+^ omitted from click-it reaction.

mTORC1 activation in the starvation-induced prevention of inflammatory damage manifested in a particularly strong phosphorylation of ribosomal protein S6 (see Figure 2B), suggesting that protein synthesis was upregulated during starvation-induced protection. To test this possibility, we studied global protein synthesis in hepatocytes metabolically labeled with cysteine/methionine derivatives modified with an alkyne moiety for later staining via click-it chemistry and detection by flow cytometric analysis. The recorded fluorescence signal specifically reflected protein synthesis, as the signal was chased by the protein synthesis inhibitor cycloheximide or by the omission of Cu^2+^, a necessary co-factor of the click-it coupling reaction (Figure 5A). In agreement with previous studies, starvation and the concomitant drop in mTORC1 activity (Figure 2B) or acute mTORC1 inhibition with rapamycin caused a sharp reduction in protein synthesis (Figure 5B) [31,40,41]. Remarkably, the addition of pro-inflammatory cytokines restored the rate of protein synthesis in starved hepatocytes, in line with the pattern of mTORC1 activity (see Figure 2B above). To confirm these data with an alternative approach, we analyzed phosphorylation of the translation initiation factor eIF2α as an independent readout for protein synthesis. A number of different stress-activated eIF2α-kinases phosphorylate Serine51 (Ser51) of eIF2α to downregulate cellular protein synthesis [42,43]. As shown in Figure 5C and quantified in Figure 5D, eIF2α-Ser51 phosphorylation was strongly induced by starvation or inflammatory stress. This finding was not unexpected, as both energy restriction and pro-inflammatory insults can trigger a cellular stress response that includes the shutdown of protein synthesis via eIF2α-Ser51 phosphorylation [43,44,45]. Noteworthily, eIF2α-Ser51 phosphorylation did not depend on mTORC1, as it was not affected by silencing Raptor (Figure 5C). Importantly, placing the inflammatory challenge on previously starved hepatocytes led to the opposite outcome, i.e., a downregulation of eIF2α-Ser51 phosphorylation, consistent with the restored rate of protein synthesis in the protected hepatocyte presented in Figure 5A. Collectively, these results suggest that starvation rewires pro-inflammatory signals in hepatocytes to induce mTOR activation to maintain robust protein synthesis rates. We also noted that the rate of protein synthesis shown in Figure 5A,B correlated with the increased resilience of starved cells towards inflammatory damage. We conclude that sustained protein synthesis probably is an important cue, acting downstream of mTORC1, for resilience induction in hepatocytes.

### 3.6. Starvation Induces Stress Granule Formation in Dependency on mTORC1, but Stress Granules Do Not Accumulate in Protected Cells

**Figure 6:**
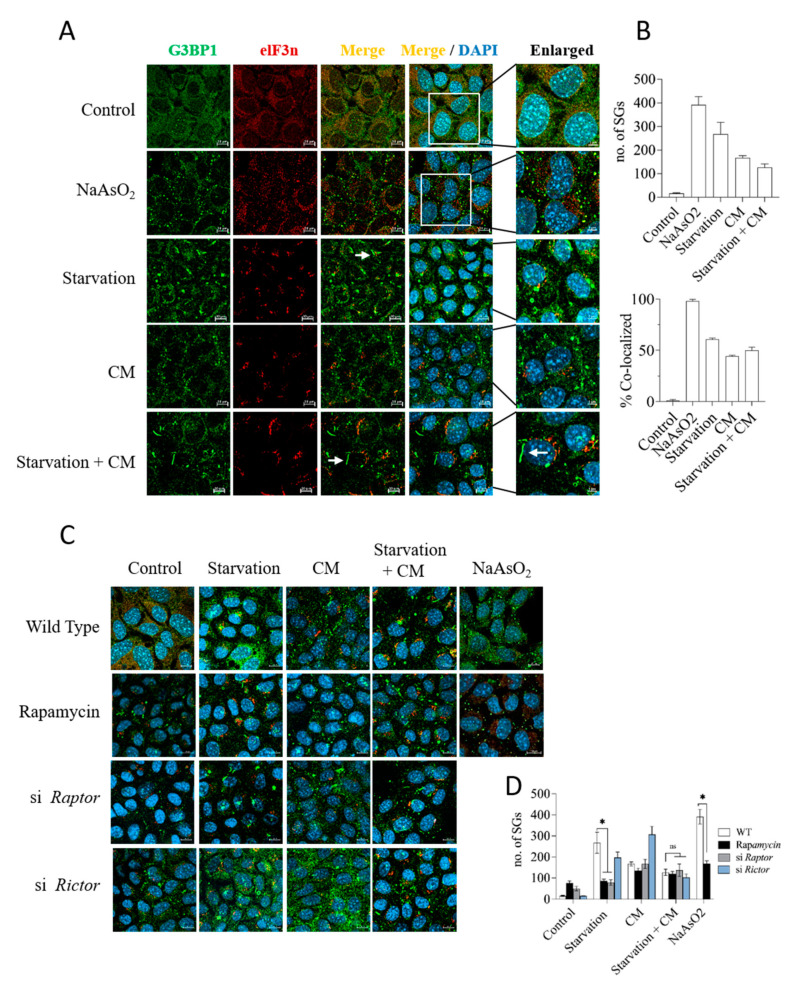
Stress granule formation in stressed hepatocytes does not correlate with protection. (**A**) Immunostaining of stress granules (SGs) using G3BP1 and elF3n antibodies in hepatocytes. NaAsO2, a potent inducer of stress granules, served as positive control. Enlarged sections are shown on the right. White arrows mark representative rod-like G3BP1-positive SG structures. (**B**) Quantification of SGs (size 0.1–2.5 µm) (upper plot) and eIF3N co-localization to G3BP1-positive SGs, plotted as % of co-localization in NaAsO2-treated cells (lower plot). Quantifications were performed with ImageJ. (**C**) SG formation in AML12 cells treated with siRNA against Raptor or Rictor or with rapamycin before being challenged with cytokines and/or starvation. Scale bar: 10 µm. (**D**) Quantification of SGs from experiment shown in panel (**C**). Data are presented as mean ± SEM; two-way analysis of variance (ANOVA) followed by Tukey’s multiple comparison test was performed; * *p* < 0.05.

Starvation, even for a period as short as 24 h, can have a serious impact on the cellular energy household, e.g., by inducing metabolic stress responses as illustrated by the engagement of AMPK and mTORC1 (see Figure 2A,B). Depletion of cellular energy stores can further trigger adaptive stress responses aimed at economizing resources while maintaining critical cellular infrastructure. One such type of adaptive stress response is the induction of stress granules, a hitherto poorly understood process in which active protein translation is stalled in stressful situations, such as energy or nutrient shortage [45,46,47]. Stress granule formation is under the control of the mTORC1 pathway [48], and the mTORC1 scaffold Raptor physically associates with stress granules [48,49,50]. With this in mind, we investigated whether stress granules are present in stressed hepatocytes and whether they may represent a component of starvation-induced mTORC1-dependent protection. Immunocytochemical staining for G3BP1, a major and regular constituent of stress granules in multiple settings [51], evidenced the formation of highly condensed G3BP1-positive puncta in AML12 hepatocytes exposed to Na-arsenite, a well-established inducer of stress granule formation (Figure 6A). A 24 h starvation regime also resulted in the robust induction of stress granules, while pro-inflammatory cytokines were much less effective in this respect (Figure 6A, quantification shown in Figure 6B). G3BP1 showed partial, but not full, co-localization with eIF3, a second prominent constituent of stress granules. This pattern has been observed in previous studies [52], supporting the specificity of the immune staining. Interestingly, we found that the condensed G3BP1-containing structures in starved cells were often elongated and rod-shaped, thus differing ostensibly from the classical speck-like stress granules (see Figure 6A). To understand if mTOR signaling was involved in stress granule formation, we monitored stress granule formation in hepatocytes after selective silencing of Raptor or Rictor. Knockdown of the mTORC1 scaffold Raptor or treatment with rapamycin caused a significant reduction in G3BP1-positive granule formation induced by starvation, whereas Rictor silencing had a mild effect that did not attain significance (Figure 6C,D). Notably, the low degree of stress granule accumulation in starved cells exposed to the pro-inflammatory insult was not sensitive to rapamycin or Raptor silencing, indicating that the stress granules were unlikely to be a protective factor in hepatocytes downstream of mTORC1.

## 4. Discussion

**Figure 7:**
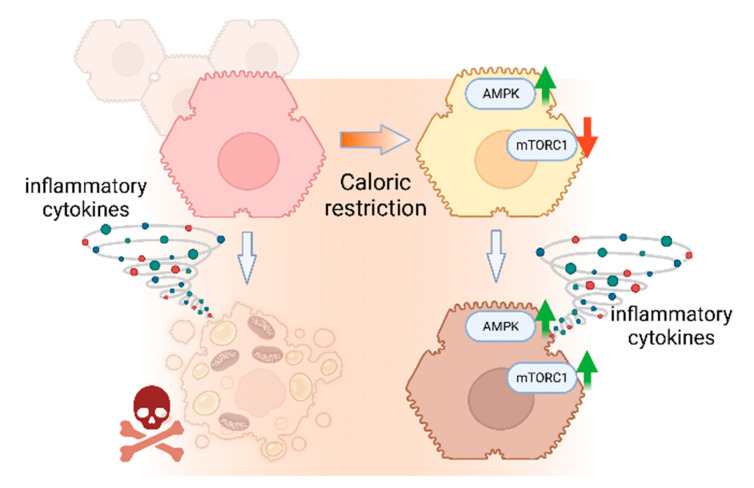
Schematic summary of current findings illustrating the protection from inflammatory damage exerted by upregulated AMPK and mTORC1 in hepatocytes exposed to caloric restriction. White arrows depict transition to inflammation. Green arrows: up-regulation; Red arrow: down-regulation. Created with Biorender.

Current therapeutic regimes in sepsis focus on fluid and hemodynamic control, combined with antimicrobial strategies. From a therapeutic point of view, however, it would be desirable to increase the resilience of organ parenchyma and thus maintain organ function during life-threatening infections. By definition, any mechanisms targeting the so-called “disease tolerance” increase parenchyma resilience without affecting the extent, course or progression of the microbial dissemination [53]. Calorie restriction has a similar effect, as it protects against severe bacterial infections in the absence of obvious effects on the bacterial load and spread [8]. However, harmful effects of calorie restriction have also been reported for selected scenarios of sepsis [54]. Noteworthily, calorie restriction markedly reduces the extent of sepsis-associated liver failure [55], while high-calorie ingestion (HCI) exacerbates sepsis-associated liver damage [9]. Our current data document a direct effect of calorie restriction on hepatocytes, protecting them from inflammatory stress-induced cell death via AMPKα and mTORC1 signaling (Figure 7). These findings therefore support the notion that the protective effect of calorie restriction in liver failure may in part result from a direct effect on the hepatocytes. In line with this possibility, we observe that the hepatocytes react promptly to starvation by activating or repressing the energy/nutrient-sensing AMPKα and mTORC1 pathways, respectively. Indeed, both pathways appear to contribute equally and be necessary to the protective effect of calorie restriction, while forced activation of either pathway is sufficient to confer protection, consistent with previous findings in other settings [15,56,57]. While the consequences of gene silencing are straightforward in the case of AMPKα1/2, given its strong activation in starvation, the pronounced effect of silencing Raptor, equaling mTORC1 knockdown, appeared counterintuitive at first, as calorie restriction reduces mTORC1 activity. However, we noticed that the mTORC1 pathway was strongly upregulated in cells exposed to the combination of starvation and inflammatory insult, making the effects of the mTORC1 functional knockdown plausible. The robust activation of mTORC1—as judged by the level of pS6K-T389 and pS6-S235/236 phosphorylation—in starved cells exposed to inflammatory cytokines was remarkable and paradoxical, as it proceeded in an apparent absence of energy signals feeding into the mTORC1 signaling machinery (the latter notion evidenced by the simultaneous high AMPKα activity, compare Figure 2A,B). 

This high mTORC1 activity in the “protected” hepatocytes was accompanied by increased protein synthesis, which was sensitive to rapamycin or Raptor knockdown. In conclusion, paradoxical mTORC1 stimulation by pro-inflammatory cytokines in starved hepatocytes translated into strong S6P phosphorylation and increased protein synthesis activity. We interpret these results to mean that starvation rewired the hepatocytes to respond to a subsequent inflammatory stimulus with protective stimulation of mTORC1 and protein translation. However, this assessment did not explain the mechanisms behind the strong and paradoxical mTORC1 activation in starved hepatocytes with low energy, low glucose and high AMPK activity.

The essential role of mTORC1 in the protection exerted by calorie restriction, as evidenced by the consequences of rapamycin treatment or Raptor knockdown, was in marked contrast to the lack of effects seen in cells with functional mTORC2 knockdown as achieved experimentally by silencing the mTORC2 scaffold Rictor. The overarching role of mTORC2 in cellular metabolism still remains debated, despite the recent identification of Akt as a central downstream target of mTORC2 [18]. Our observation that neither Rictor knockdown nor pharmacological Akt inhibition precluded the higher resilience of starved hepatocytes was in agreement with the reported linear mTORC2/Akt signaling topology. The latter finding, however, was striking as Akt commonly acts upstream of mTORC1. However, our findings show that in starved AML12 hepatocytes, the challenge with inflammatory cytokines induced mTORC1 activity in the absence of detectable Akt phosphorylation (compare Figure 2B,E). These findings underscore the paradoxical nature of the mTORC1 activation pathway that is associated with the resilient state of starved hepatocytes. Moreover, our data emphasize the markedly different functions of mTORC1 and mTORC2 in cellular (hepatocyte) metabolism.

mTORC1 is a well-established major driver of global protein synthesis. Our findings document a strong correlation of both parameters, suggesting a role for robust protein synthesis in the mTORC1-dependent induction of hepatocyte resilience. Another major stress adaptive pathway regulated by mTORC1 is autophagy. Autophagy has previously been implicated in the induction of disease tolerance and protection against sepsis-associated organ failure and mortality in preclinical models [33,58]. However, the fundamental role of autophagy in organ failure remains dubious and obscure, as other studies reported deleterious effects of autophagy on tissue resilience and recovery in sepsis [59,60]. Our findings do not support a role for autophagy in the starvation-induced protection of isolated hepatocytes. Given the strong cross-talk and interdependence among organs during the host response to severe infections [61], these findings can be rationalized by assuming that hepatocyte autophagy serves other tissues’ welfare rather than hepatocyte protection itself. The liver reacts to stress by inducing a particularly high level of autophagy [62]. Indeed, autophagy was first described in the 1960s in rodent liver [63]. Liver autophagy is important for supplying other tissues with nutrients and metabolic building blocks in times of need. This may have led to an overarching function of hepatic autophagy, aimed at ensuring the supply of nutrients to other tissues [64], rather than conferring resilience to hepatocytes themselves. It is tempting to speculate that this behavior, based on the distinct wiring of intracellular metabolic signaling of hepatocytes in the starvation state, may reflect an effort to distribute resources among organs and tissues in a way that best ensures the fitness of the whole organism [65].

Similar considerations may apply to the process of stress granule formation, which we document here for the first time in hepatocytes subjected to inflammatory stress. Stress granules are considered to represent phase-separated aggregates of stalled protein synthesis platforms. They have been documented and studied mainly in the context of neurodegeneration [66] and cancer, where they sometimes exert a strong pro-survival effect [67]. However, little is known about their role in inflammation and infection-associated tissue damage [68]. In our experiments stress granule levels do not correlate with hepatocyte survival, even though caloric restriction itself did induce a marked formation of unconventionally shaped stress-granule-like aggregates. Whether stress granule genesis in stressed hepatocytes serves primarily the survival of the hepatocytes or the provision of services to other organs and tissues will have to await further investigation.

In summary, the findings presented herein partially recapitulate data from preclinical animal models in cultured hepatocytes, illustrating that calorie restriction increases the resilience of hepatocytes towards inflammatory stress. Protection highly depends on AMPKα activity and on mTORC1 signaling. The latter pathway was paradoxically activated in the starved energy-depleted hepatocytes, driving protein synthesis, in what seemed to be a dedicated mechanism of tissue resilience. These results provide solid evidence that adaptation to inflammatory stress in hepatocytes can occur through a readjustment of the intracellular metabolic circuitry. Finally, our study shows how working with untransformed cell lines can contribute to deciphering molecular mechanisms of stress adaptation and resilience acquisition in parenchymal cells.

## Data Availability

All raw data and study materials will be made available by the authors upon reasonable request.

## Abbreviations

CM: cytokine and LPS mix; CR: calorie restriction; SG: stress granule.
