# Correction: Hussain et al. Starvation Protects Hepatocytes from Inflammatory Damage through Paradoxical mTORC1 Signaling. *Cells* 2023, *12*, 1668

**DOI:** 10.3390/cells13181570

**Published:** 2024-09-19

**Authors:** Iqra Hussain, Harini K. Sureshkumar, Michael Bauer, Ignacio Rubio

**Affiliations:** 1 Department for Anesthesiology & Intensive Care Medicine, Jena University Hospital, Member of the Leibniz Center for Photonics in Infection Research (LPI), 07747 Jena, Germany; 2 Integrated Research and Treatment Center, Center for Sepsis Control and Care, Jena University Hospital, 07747 Jena, Germany

## Error in Figure 3B

In the original publication [1], there was an error in the published Figure 3B. Due to an unintentional and unnoticed drag-and-drop procedure, the panels “wild-type/CM” and “wild-type/rapamycin” were duplicated. This error was noticed by the authors when reviewing the final online version of the manuscript. The corrected Figure 3 appears below.

**Figure 3:**
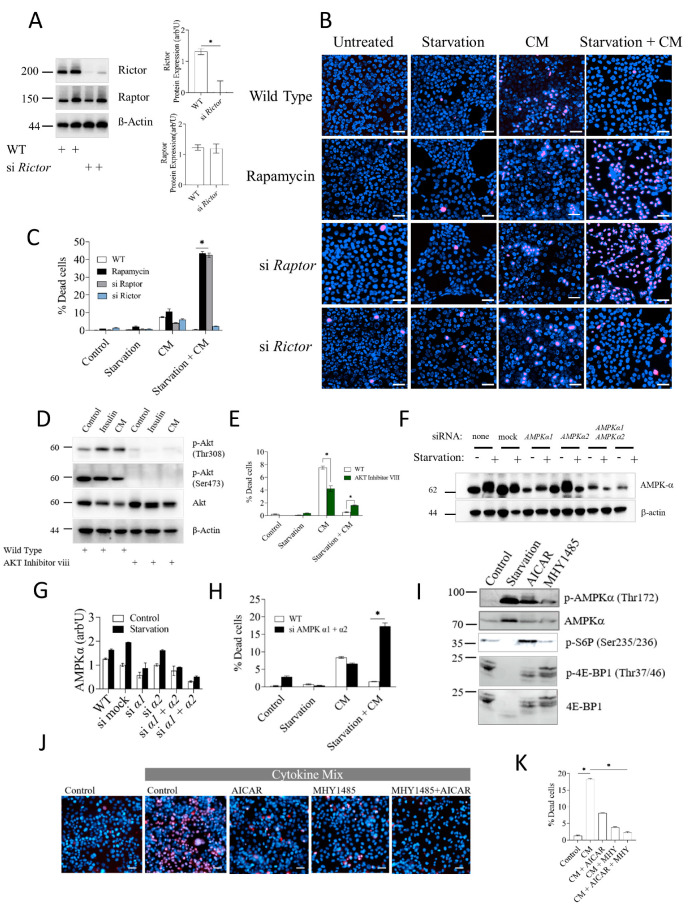
Protection against inflammatory damage provided by calorie restriction requires mTORC1 and AMPKα. (**A**) Western blot of Raptor and Rictor protein levels in cells subjected to siRNA-mediated *Rictor* knockdown. Quantification of Rictor protein levels normalized to β-actin with ImageJ is shown on the right plot. (**B**) Cell death assay in AML12 cells that underwent siRNA-mediated knockdown of *Raptor* or *Rictor*. Inflammatory stress and starvation treatments were as before. Cell death was evaluated by dual PI/Hoescht33342 live/dead staining. Scale bar: 50 µm. (**C**) Quantification of data shown in B. (**D**) Potency of Akt inhibitor VIII in AML12 cells. AML12 cells treated with 10 μM Akt inhibitor VIII for 24 h or left untreated were stimulated with insulin or cytokine mix and processed for immunodetection by Western blotting of activated/phosphorylated and total Akt with the indicated antibodies. (**E**) Cell death assay of AML12 cells treated with 10 μM Akt inhibitor VIII for 24 h or left untreated prior to exposure to starvation and/or inflammatory cytokines. (**F**) AML12 cells transfected with siRNAs against mock, AMPKα1 and/or AMPKα2 were exposed or not to overnight starvation (−/+) followed by Western blot detection of total AMPKα. None: no transfection mix or siRNA; Mock: transfection mix with control siRNA with no homology to any known gene sequence. (**G**) Densitometric quantification of AMPKα levels shown in panel (**F**). (**H**) Cell death assay of AML12 cells transfected with a mix of siRNAs against AMPKα1 and AMPKα2 and challenged by starvation and/or inflammatory cytokines as indicated. (**I**) AML12 cells were treated with AICAR or MHY1485, and cell extracts were subjected to Western blot detection with the indicated antibodies. (**J**) AML12 cells were treated with AICAR or MHY1485 and exposed to cytokine mix as indicated. Cell death was measured as before. (**K**) Quantification of cell death data in panel (**J**). Cell death data are presented as mean ± SEM; two-way analysis of variance (ANOVA) followed by Tukey’s multiple comparison test was performed. * indicates *p* < 0.05, ns indicates non-significance.

The authors state that the scientific conclusions are unaffected. This correction was approved by the Academic Editor. The original publication has also been updated.
